# Ring1a protects against colitis through regulating mucosal immune system and colonic microbial ecology

**DOI:** 10.1080/19490976.2023.2251646

**Published:** 2023-09-01

**Authors:** Yashu Wang, Qianru Li, Jiayu Zhang, Pingping Liu, Huaixin Zheng, Lijuan Chen, Zhen Wang, Chen Tan, Min Zhang, Hongxia Zhang, Wenqing Miao, Yuke Wang, Xiaoyan Xuan, Guoqiang Yi, Peng Wang

**Affiliations:** a Department of Microbiology and Immunology, School of Basic Medical Sciences, Zhengzhou University, Zhengzhou, China; b Infection, Inflammation and Immunity Center, the Academy of Medical Sciences of Zhengzhou University, Zhengzhou, China; c Lab of Pathology, Hebei Medical University, Shijiazhuang, China; d Shenzhen Branch, Guangdong Laboratory of Lingnan Modern Agriculture, Key Laboratory of Livestock and Poultry Multi-omics of MARA, Kunpeng Institute of Modern Agriculture at Foshan, Agricultural Genomics Institute at Shenzhen, Chinese Academy of Agricultural Sciences, Shenzhen, China

**Keywords:** Ring1a, DSS colitis, mucosal immune system, intestinal microbiota, scRNA-seq

## Abstract

Inflammatory bowel disease (IBD) represents a prominent chronic immune-mediated inflammatory disorder, yet its etiology remains poorly comprehended, encompassing intricate interactions between genetics, immunity, and the gut microbiome. This study uncovers a novel colitis-associated risk gene, namely Ring1a, which regulates the mucosal immune response and intestinal microbiota. Ring1a deficiency exacerbates colitis by impairing the immune system. Concomitantly, Ring1a deficiency led to a *Prevotella* genus-dominated pathogenic microenvironment, which can be horizontally transmitted to co-housed wild type (WT) mice, consequently intensifying dextran sodium sulfate (DSS)-induced colitis. Furthermore, we identified a potential mechanism linking the altered microbiota in Ring1aKO mice to decreased levels of IgA, and we demonstrated that metronidazole administration could ameliorate colitis progression in Ring1aKO mice, likely by reducing the abundance of the *Prevotella* genus. We also elucidated the immune landscape of DSS colitis and revealed the disruption of intestinal immune homeostasis associated with Ring1a deficiency. Collectively, these findings highlight Ring1a as a prospective candidate risk gene for colitis and suggest metronidazole as a potential therapeutic option for clinically managing *Prevotella* genus-dominated colitis.

## SUMMARY

We found that PcG protein Ring1a could be a new risk gene for colitis. Ring1a deficiency causes aggravated colitis by regulating the mucosal immune system and colonic microbial ecology.

## Introduction

Inflammatory bowel disease (IBD) is a chronic immune-mediated relapsing inflammatory disorder of the intestine and is recognized as a prevalent gastrointestinal condition worldwide.^1,2^ Despite its high prevalence, the etiology of IBD remains inadequately understood. Existing evidence strongly suggests that the pathophysiology of IBD arises from intricate interactions among environmental, microbial, and immune-mediated factors in individuals with a genetic susceptibility.^3–5^ Two prominent clinical phenotypes of IBD are Crohn’s disease (CD) and ulcerative colitis (UC). Although CD and UC exhibit distinct characteristics, both disorders share a common pathogenesis involving dysbiosis of symbiotic microorganisms and intestinal inflammation.^4^ The mammalian gastrointestinal tract harbors a complex microbial community comprised of trillions of bacteria, which dynamically interact and co-evolve with the host immune system.^6^ The resident gut symbionts play fundamental roles in host physiological processes, nutrition and metabolism, immune system development, and defense against pathogen colonization.^7,8^ In recent years, extensive research has focused on unraveling the intricate interplay between the immune system and the gut microbiota, as well as the impact of the intestinal microbiota on immune system functioning in various diseases, including IBD.^6^ Therefore, understanding and uncovering the complex relationships between the intestinal microbiota and the host immune system holds promise for elucidating the pathogenesis of IBD and providing novel preventive and therapeutic strategies.

Polycomb group (PcG) proteins comprise a class of epigenetic repressors that catalyze histone modifications involved in gene regulation, cell differentiation, and development.^9,10^ These proteins mainly form two protein complexes, namely polycomb repressive complexes 1 (PRC1) and 2 (PRC2).^11^ Generally, PRC1 acts as a transcriptional repressor, inhibiting gene transcription through catalyzing histone H2A monoubiquitylation at lysine 119 (H2AK119ub1).^12–14^ However, several studies have revealed that PRC1 can also function as a transcriptional activator, promoting gene transcription.^15,16^ Mammalian PRC1 comprises the E3 ubiquitin ligase Ring1a, Ring1b, and one of six PCGF proteins. The E3 ubiquitin ligase Ring1a or its homolog Ring1b constitutes the catalytic core of PRC1. Ring1a exerts overlapping functions with Ring1b in catalyzing H2AK119ub1.^12^ By cooperating with Ring1b and other PcG proteins, Ring1a regulates processes such as chromosome X inactivation,^12^ Hox gene silencing,^17^ and ES and AML stem cell identity maintenance.^18–20^ Although Ring1a is widely expressed in various immunocyte types, its role in the immune system and immune-related disorders remains largely unexplored. Ring1a has been reported to play an essential role in converting T cells to B cells. T cell deficiency of Ring1a and Ring1b leads to overexpression of PAX5, which hampers their maturation and promotes their conversion to B cells.^21^ Moreover, Ring1a has been shown to bind to the *Il4* and *Ifng* loci in differentiated T helper cells.^22^ However, the involvement of Ring1a in chronic immune-mediated IBD remains uninvestigated.

In this study, we investigated the contribution of the polycomb complex protein Ring1a to colitis development and revealed how Ring1a regulates colitis through the immune system and intestinal microbiota. We found that Ring1a deficiency exacerbated colitis exacerbation by perturbing the immune system and altering the gut microbiota composition. Additionally, we demonstrated that colitis exacerbations caused by Ring1a deficiency could be transmitted to co-housed wild type (WT) mice and persist over time. Furthermore, 16S rRNA sequencing revealed that Ring1a deficiency led to a *Prevotella* genus-dominated microenvironment, potentially attributable to reduced IgA production. Additionally, we discovered that metronidazole administration mitigated colitis exacerbations induced by Ring1a deficiency. Furthermore, through Single-cell RNA sequencing (scRNA-seq) transcriptional analysis, we characterized the immune landscape of intestinal lamina propria (LP) in dextran sodium sulfate (DSS)-induced colitis and uncovered the disruption of intestinal immune homeostasis associated with Ring1a deficiency.

## Materials and methods

### Mice

CD45.1^+^ mice were purchased from Beijing University Experimental Animal Center (Beijing, China). Ring1a knockout (Ring1aKO) mice (Saiye, Guangzhou, China) were crossed with C57BL/6 J (B6) (Huafukang Bioscience, Beijing, China). All mice were bred and maintained under specific pathogen-free conditions in the animal facility of Zhengzhou University. Sex-matched littermate WT and Ring1aKO mice of 8 to 12- week were used for experiments. For co-housing experiments, 5 to 6-week and gender-matched littermate WT and Ring1aKO mice were co-housed for at least 2 to 4 weeks. All animal studies were approved by the Animal Ethics Committee of Zhengzhou University (Zhengzhou, China).

### DSS colitis

Mice were treated with 2.5% (w/v) DSS (M.W. = 36,000–50,000 Da; MP Biomedicals) dissolved in drinking water for 7 or 8 consecutive days, and then mice were sacrificed. Ring1a inhibitor (PRT4165, 8 mg/kg, Sigma-Aldrich) or the same dose of dimethyl sulfoxide was intraperitoneally injected into mice daily from day −2 to day 3 during DSS-induced colitis model to evaluate the effect of Ring1aKO inhibitor on mouse colitis. Body weight and disease activity index (DAI) were assessed daily. DAI scores were calculated according to weight loss, stool consistency, and stool blood content/rectal bleeding, as previously described.^23^

### Histopathology

Colon tissues were fixed in 4% paraformaldehyde and then embedded in paraffin. Subsequently, 5 μm sections were stained with hematoxylin and eosin (H&E).^24^ Histological sections were scored as previously described.^23^ Epithelium: normal morphology (0), loss of goblet cells (1), loss of goblet cells in large areas (2), loss of crypts (3), and loss of crypts in large areas (4); and infiltration: no infiltrate (0), infiltrate around crypts (1), infiltrate reaching the lamina muscularis mucosae (2), extensive infiltration reaching the lamina muscularis mucosae and thickening of the mucosa (3), and infiltration of the submucosal layer (4). A total histological score equals to the sum of both scores.

### Isolation of colonic LP cells

Colonic LP cells were isolated as previously described.^25^ Briefly, colons were sheared longitudinally and rinsed with PBS, and then were incubated in PBS (5 mM EDTA) on the shaker for 12 min at 37°C. Colon tissues were washed and minced, and then digested in 5 mL of 1640 medium containing 10% FBS, 1 mg/mL Type IV collagenase, and 40 µg/mL DNase I at 220 rpm for 60 min at 37°C. After digestion, cells were filtered successively through 75-µm and 37-µm filter membranes.

### Flow cytometry

For analysis of surface markers, prepared single cell suspension (100 μL, 5 × 10^6^ cells/ml) was stained with anti-mouse CD45 (30-F11, Biolegend), anti-mouse CD19 (6D5, Biolegend), anti-mouse CD45.1 (A20, Tonbo), anti-mouse CD45.2 (104, Invitrogen), anti-mouse IgA (mA-6E1, Invitrogen). Stained cells were analyzed on FACSCelesta (BD Biosciences) and the obtained data were analyzed with FlowJo_V10 software.

### Cell Sorting by Flow Cytometry

4 × 10^6^ colonic LP cells were suspended in 300 μL PBS. Firstly, incubated with anti-mouse CD16/32 antibody for 10 min, and then incubated with anti-mouse CD45 antibody for 30 min. After being washed with cold PBS, cells were incubated with 7-aminoactinomycin (7AAD) for 10 min. Lastly, CD45^+^7AAD^−^ cells were sorted on BD FACSMelody.

### 16S rRNA analysis

Fecal samples were collected separately from single-housed and co-housed mice (2 weeks) into sterile tubes before DSS treatment, and were rapidly snap-frozen in liquid nitrogen. The total DNA from fecal bacteria was extracted and 16S rRNA genes (V3-V4 region) were amplified using primers (F: 5’-ACTCCTACGGGAGGCAGCA-3’, R: 5’-GGACTACHVGGGTWTCTAAT-3’). Sequencing was performed using the Illumina NovaSeq platform (PANOMIX Biomedical, Suzhou). Data were analyzed using QIIME2. Taxonomy was assigned to ASVs using the classify-sklearn naïve Bayes taxonomy classifier in the feature-classifier plugin against the SILVA Release 132 database.

### Depletion of intestinal microbiota

WT and Ring1aKO mice were gavaged with a combination of four antibiotics (ampicillin 100 mg/kg, vancomycin 50 mg/kg, neomycin sulfate 100 mg/kg and metronidazole 100 mg/kg) to deplete bacteria or amphotericin B (1 mg/kg) to deplete fungi for 14 consecutive days (2 times/day). Before and during DSS-induced colitis model, WT and Ring1aKO mice were gavaged with metronidazole (100 mg/kg) for every 12 hours consecutive 14 days (from day −10 to day 4), feces were collected for 16S rRNA sequencing at day 0.

### Bone marrow transplantation

For full bone marrow chimeras, 8 to 10- week male WT and Ring1aKO mice were sublethally irradiated with 9 Gy, and then received 1 × 10^7^ WT or Ring1aKO bone marrow cells 4 hours later. For mixed bone marrow chimeras, 8 to 10-week male CD45.1 mice were sublethally irradiated with 9 Gy and received 1 × 10^7^ WT and Ring1aKO bone marrow cells (1:1) 4 hours later.

### scRNA-seq and data analysis

Intestinal LP immune cells were prepared and pooled for scRNA-seq from colon tissues of five mice of each group (DSS-treated WT and Ring1aKO mice). Sorted CD45^+^ cells were loaded onto the Chromium microfluidic chips and barcoded with a 10× Chromium Controller to generate single-cell gel beads-in-emulsions (GEMs). The scRNA-seq libraries were constructed using Single Cell 3’ Library and Gel Bead Kit V3.1 (10× Genomics). The libraries were finally sequenced using the Illumina Novaseq 6000 platform with a sequencing depth of at least 100,000 reads per cell with a pair-end 150 bp (PE150) reading strategy. Raw sequence data were mapped to the mm10 genome reference using CellRanger-count (3.0.1) to produce feature-barcode matrices. The Seurat V3.0 R package (https://satijalab.org/seurat) was used to perform data filtration, sample integration, gene normalization, dimension reduction and data visualization. Samples including WT and Ring1aKO were integrated as one object by Seurat “IntegrateData” function. It used t-Distributed Stochastic Neighbor Embedding (t-SNE) to visualize single-cell clusters, employing the top 25 principal components with the largest variance (at resolution = 0.7 for all the merged samples). Calculate the percent of mitochondrial (“percent.mito”) and ribosome (“percent.ribo”)-related genes for each cell, and filter out the cells with a value of “percent.mito” less than 5, and the value of “percent.ribo” less than 40, respectively. Differentially expressed genes (DEGs) of each cluster under the threshold of adjusted *P* < .05 (corrected *P* value from T-test by Benjamini-Hochberg correction) and LogFoldChang ≥ 0.5 compared to other clusters by Seurat’s “FindMarkers” function. The scRNA-seq data has been submitted to the GEO database (GSE210866, https://www.ncbi.nlm.nih.gov/geo/query/acc.cgi?acc=GSE210866).

### Statistical analysis

All data were presented as mean ± SD. Unpaired two-tailed Student’s t-test was used for data from two groups. Data from more than two groups were subjected to a one-way analysis of variance (ANOVA) with SAS 9.2 version. Comparisons were considered statistically significant when the *P* value was less than 0.05. * *P* < .05, ** *P* < .01.

## Results

### Ring1a-deficient mice developed severe colitis

**Figure 1.**
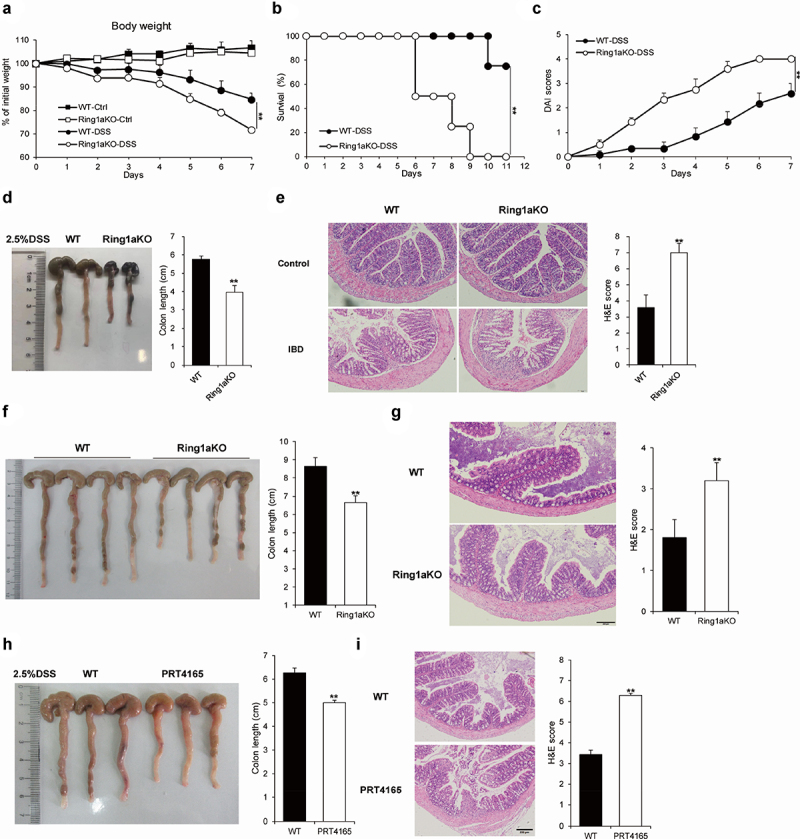
Ring1a deficiency aggravated colitis.

The sulfated polysaccharide DSS-induced colitis is a commonly used model to simulate the pathological features of IBD.^26^ Therefore, we employed this model to investigate the role of Ring1a in colitis. During the process, Ring1aKO mice exhibited significant weight loss in the late stages (Figure 1a) and a higher mortality rate than that of WT mice (Figure 1b). Moreover, Ring1aKO mice exhibited remarkably higher disease activity index (DAI) scores than those of WT mice (Figure 1c). Furthermore, the colon length of Ring1aKO mice was considerably shorter than that of WT mice (Figure 1d). Consistently, the colon tissues of Ring1aKO mice were also infiltrated with more inflammatory cells and showed more severe intestinal damage than those of WT mice (Figure 1e). Furthermore, we found that Ring1aKO mice developed spontaneous colitis as they aged. One-year-old Ring1aKO mice displayed remarkably shorter colon length and more severe inflammatory infiltration than those of age-matched WT mice of the same age (Figures 1f–g). To further verify the role of Ring1a in colitis, we investigated the effect of a Ring1a inhibitor, PRT4165, in the DSS-induced colitis model. The results showed that PRT4165-treated mice exhibited shortened colon length (Figure 1h), increased histological inflammatory infiltrates, and exacerbated intestinal damage (Figure 1i). Notably, the effect of the Ring1a inhibitor on colitis appeared to be less than that of the Ring1a deficiency. These findings collectively indicate that Ring1a deficiency or inhibition exacerbates DSS-induced colitis, suggesting a protective role of Ring1a in colitis and its negative regulatory function in this condition.

(a) The body weight of WT and Ring1aKO mice under normal condition and DSS treatment. The survival rate (b) and DAI scores (c) of WT and Ring1aKO mice in DSS-induced colitis (*n* = 4). (d) Colon lengths of WT and Ring1aKO mice in DSS-induced colitis (*n* = 4). (e) H&E staining and H&E scores of colon tissues of WT and Ring1aKO mice under normal conditions and DSS treatment. (f) Colon lengths of aged WT and Ring1aKO mice under normal conditions (*n* = 5). (g) H&E staining and H&E scores of colon tissues of aged WT and Ring1aKO mice under normal conditions. (h) Colon lengths of WT mice treated with or without Ring1a inhibitor PRT4165 in DSS-induced colitis (*n* = 4). (I) H&E staining and H&E scores of colon tissues of WT mice treated with or without Ring1a inhibitor PRT4165 in DSS-induced colitis. Data are shown as mean ± SD, representing one of at least three independent experiments. *P < .05, **P < .01.

### Ring1a deficiency intrinsically aggravates DSS-induced colitis in immune cells

**Figure 2.**
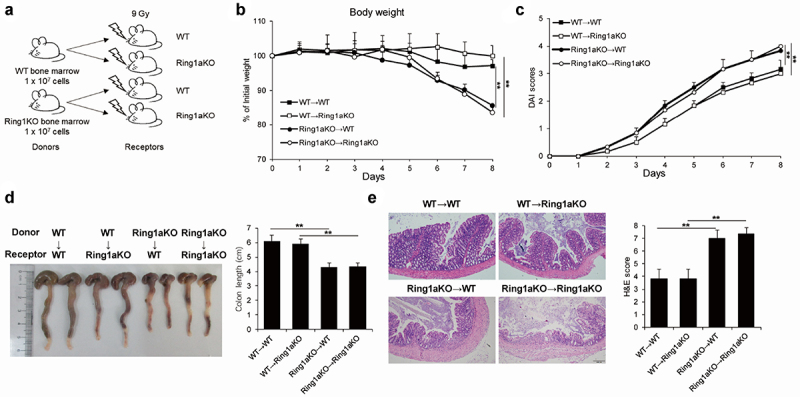
Ring1a deficiency in immune cells intrinsically aggravates DSS induced colitis.

To elucidate whether the aggravated colitis resulting from Ring1a deficiency is primarily attributable to the effects of immune cells or other cell types, we performed reciprocal bone marrow transfer experiments between WT and Ring1aKO mice. Compared to WT or Ring1aKO recipient mice that received WT bone marrow cells (designated as WT→WT and WT→Ring1aKO), WT and Ring1aKO recipient mice that received Ring1aKO bone marrow cells (designated as Ring1aKO→WT and Ring1aKO→Ring1aKO) exhibited significantly more body weight loss, a higher DAI score, and shorter colon length (Figures 2a–d). Furthermore, colon tissues from Ring1aKO→WT and Ring1aKO→Ring1aKO mice exhibited more severe histological inflammatory infiltration and intestinal damage than those from WT→WT and WT→Ring1aKO mice (Figure 2e). Conversely, no considerable differences were observed between WT→WT and WT→Ring1aKO mice with respect to the indicators of colitis (Figures 2a–e). Hence, Ring1a deficiency aggravates colitis in the DSS-induced colitis model through its effects onimmune cells.

(a) The schematic plot of construction of bone marrow chimera. The body weight (b) and DAI scores (c) of irradiated WT and Ring1aKO mice transferred with WT or Ring1aKO bone marrow in DSS-induced colitis. (d) Colon lengths of irradiated WT and Ring1aKO mice transferred with WT or Ring1aKO bone marrow in DSS-induced colitis. (e) H&E staining and H&E scores of irradiated WT and Ring1aKO mice transferred with WT or Ring1aKO bone marrow in DSS-induced colitis. Data are shown as mean ± SD (*n* = 4-5), representing one of at least three independent experiments. *P < .05, **P < .01.

### Ring1a deficiency leads to colitis exacerbation mediated by the transferable intestinal microbiota

Prior studies have unequivocally established the critical role of intestinal microbiota in colitis pathogenesis, demonstrating that genetically modified mice exhibited altered susceptibility to colitis due to changes in gut microbiota composition.^3,27–29^ To investigate the functional relationship between the intestinal microbiota and Ring1a deficiency-induced colitis exacerbation in the DSS-induced colitis model, we first co-housed adult Ring1aKO mice with age- and sex-matched WT mice for two weeks and then conducted DSS-induced colitis. Remarkably, we found that co-housed WT and Ring1aKO mice exhibited comparably severe DSS-induced colitis, with a tendency for greater severity in WT mice co-housed with Ring1aKO mice (Supplementary Figure S1).

**Figure 3.**
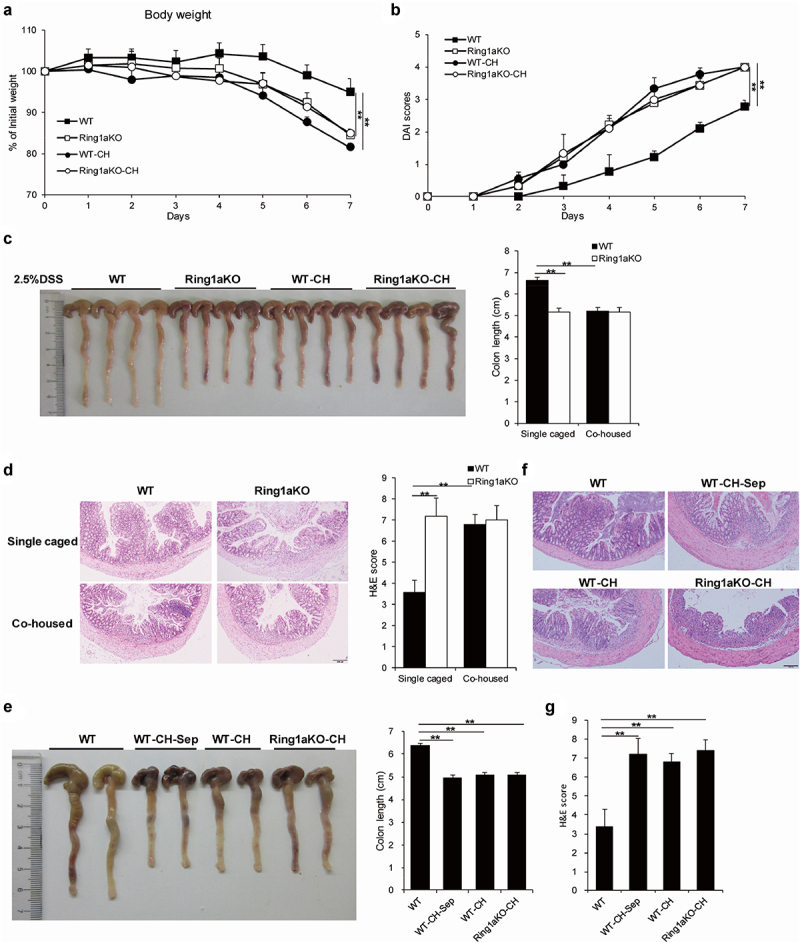
Exacerbation of colitis caused by Ring1a deficiency owing to intestinal microbiota that is transferable to co-housed WT mice.

Subsequently, to verify our speculation, we simultaneously subjected single-housed WT and Ring1aKO mice, as well as co-housed WT (designated as WT-CH) and Ring1aKO mice (designated as Ring1aKO-CH), to DSS-induced colitis. Notably, WT-CH mice demonstrated equally severe DSS-induced colitis compared to Ring1aKO-CH mice and single-housed Ring1aKO mice. The results showed all three groups (single-house Ring1KO, WT-CH, and Ring1aKO-CH) exhibited significantly greater bodyweight loss, higher DAI scores, shorter colon lengths, and more pronounced histological inflammatory infiltration and intestinal damage compared to single-housed WT mice (Figures 3a–d). Importantly, no significant differences were observed in these colitis indicators among single-housed Ring1aKO mice, WT-CH mice, and Ring1aKO-CH mice (Figures 3a–d). These results conclusively demonstrate that the variations in colitis severity observed among single-housed WT and Ring1aKO mice are indeed driven by differences in their respective intestinal microbiota. Additionally, we provide evidence that the intestinal microbiota of Ring1aKO mice possesses increased pathogenicity and can be transferred to co-housed WT mice.

The body weight (a) and DAI scores (b) of single-housed and co-housed WT and Ring1aKO mice in DSS-induced colitis. (c) Colon lengths of single-housed and co-housed WT and Ring1aKO mice in DSS-induced colitis. (d) H&E staining and H&E scores of single-housed and co-housed WT and Ring1aKO mice in DSS-induced colitis. (e) Colon lengths of single-housed WT, WT-CH-Sep, and co-housed WT and Ring1aKO mice in DSS-induced colitis. H&E staining (f) and H&E scores (g) of single-housed WT, WT-CH-Sep, and co-housed WT and Ring1aKO mice in DSS-induced colitis. Data are shown as mean ± SD (*n* = 5), representing one of at least three independent experiments. *P < .05, **P < .01.

To investigate whether the effect of the transferable pathogenic intestinal microbiota from Ring1aKO mice to co-housed WT mice can be sustained over an extended period of time, a group of age- and sex-matched WT mice were co-housed with Ring1aKO mice for two weeks and then separated for four weeks (designated as WT-CH-Sep) before colitis induction. Intriguingly, WT-CH-Sep mice exhibited significantly shorter colon length, a more severe histological inflammatory infiltrate, and greater intestinal damage than those exhibited by single-housed WT mice (Figures 3e–g). Notably, there were no differences in colon length, histological inflammatory infiltrate, or intestinal damage among WT-CH-Sep, WT-CH, and Ring1aKO-CH mice (Figures 3e–g). Therefore, these findings demonstrate that the effect of the transferable pathogenic intestinal microbiota from Ring1aKO mice to co-housed WT mice can persist for at least one month.

### Gut bacteria caused severe colitis in Ring1aKO mice

**Figure 4.**
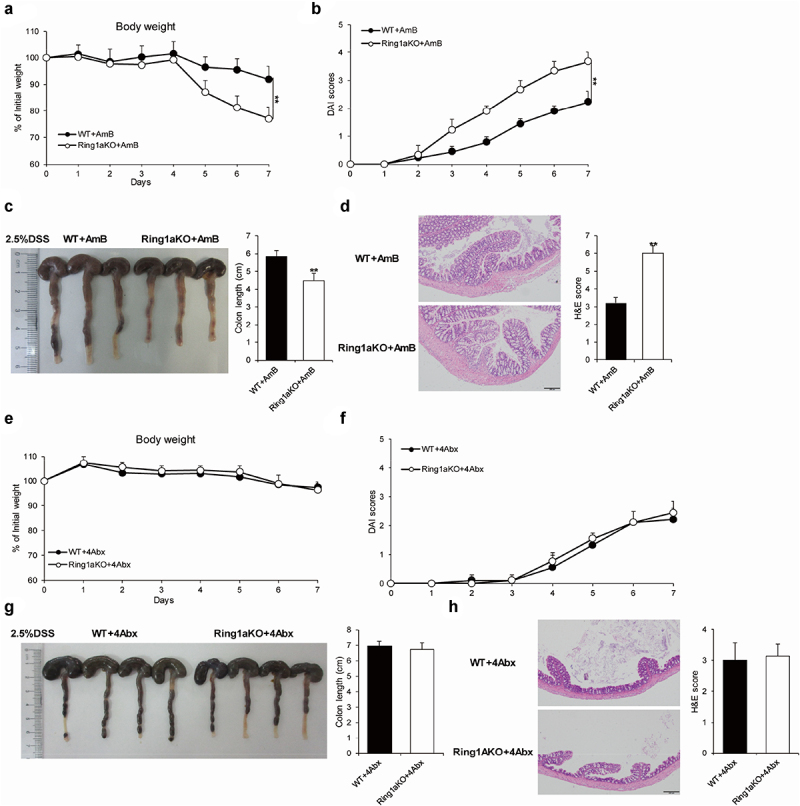
Gut bacteria caused severe colitis in Ring1aKO mice.

The gut microbiota comprises trillions of bacteria and fungi.^30^ To identify whether increased colitis severity is driven by gut bacteria or fungi, single-housed WT and Ring1aKO mice were orally administered a combination of four antibiotics (ampicillin, vancomycin, metronidazole, and neomycin) or amphotericin B for two weeks, which are known to effectively eliminate gut bacteria or fungi, respectively.^27,31^ The results showed that removing fungi using amphotericin B did not alter the severity of DSS-induced colitis in Ring1aKO mice. Even after treatment with amphotericin B, Ring1aKO mice experienced greater weight loss, shorter colon length, and more severe histological inflammation than those experienced by WT mice (Figures 4a–d). Conversely, colitis severity was comparable between WT and Ring1aKO mice when gut bacteria were eliminated using a combination of four antibiotics. Furthermore, Ring1aKO mice exhibited similar weight loss, colon length, and histological inflammation to WT mice following antibiotic treatment (Figures 4e–h). Thus, it is evident that gut bacteria, rather than fungi, contribute to the development of severe colitis in Ring1aKO mice.

The body weight (a) and DAI scores (b) of WT and Ring1aKO mice treatment with AmB in DSS-induced colitis (*n* = 4-5). (c) Colon lengths of WT and Ring1aKO mice treatment with AmB in DSS-induced colitis (*n* = 4-5). (d) H&E staining and H&E scores WT and Ring1aKO mice treatment with AmB in DSS-induced colitis. The body weight (e) and DAI scores (f) of WT and Ring1aKO mice treatment with 4Abx in DSS-induced colitis (*n* = 5). (g) Colon lengths of WT and Ring1aKO mice treatment with 4Abx in DSS-induced colitis (*n* = 5). (h) H&E staining and H&E scores WT and Ring1aKO mice treatment with 4Abx in DSS-induced colitis. Data are shown as mean ± SD, representing one of at least three independent experiments. *P < .05, **P < .01.

### Ring1a deficiency alters the composition of intestinal bacteria, likely due to decreased lgA production

**Figure 5.**
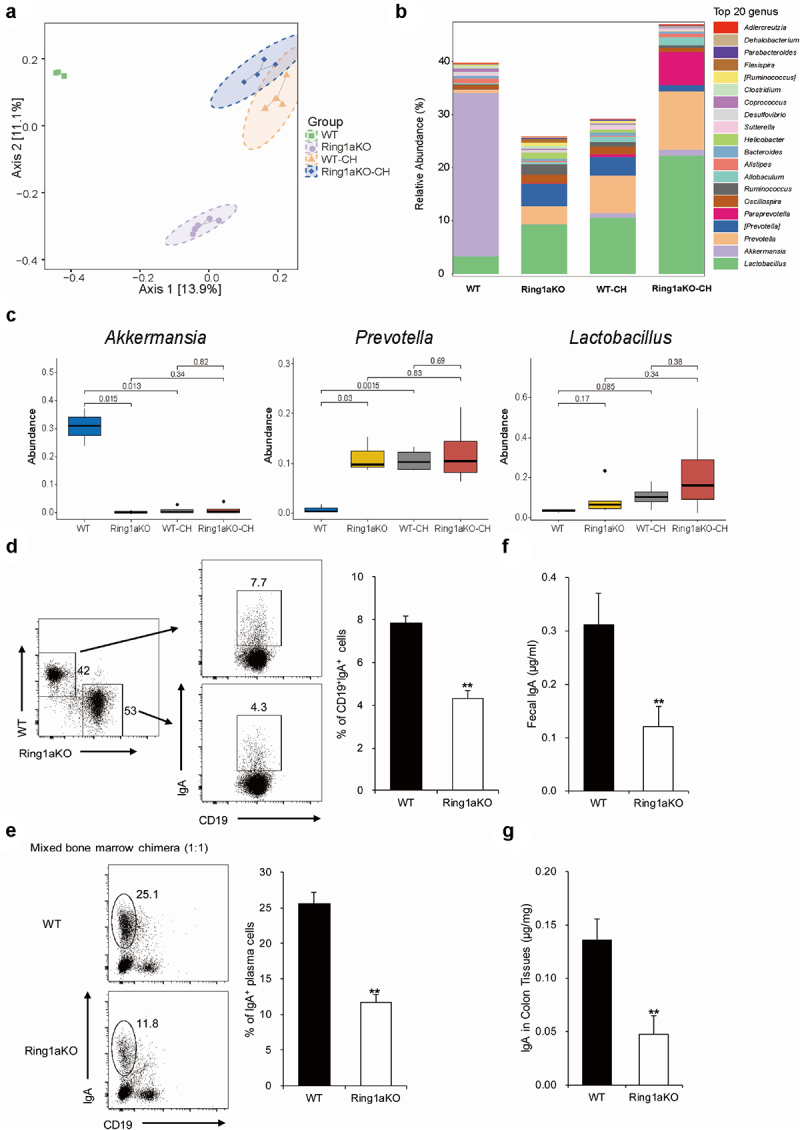
Ring1a deficiency altered the composition of intestinal bacteria decreased the production of lgA.

To further identify the specific colitogenic bacteria responsible for the exacerbated colitis in Ring1aKO mice, we collected fecal samples from single-housed WT, Ring1aKO, and co-housed WT and Ring1aKO mice for 16S rRNA sequencing. Cluster analysis revealed distinct differences in the fecal bacterial phylogenetic architecture between single-housed WT and Ring1aKO mice (Figure 5a). However, after two weeks of co-housing, WT-CH and Ring1aKO-CH mice displayed similar fecal bacterial phylogenetic architectures (Figure 5a). Furthermore, the bacterial composition of the fecal microbiota in WT-CH and Ring1aKO-CH mice resembled that of single-house Ring1aKO mice, which was different from that of single-housed WT mice (Figure 5b). Interestingly, the bacterial component showed that *Akkermansia* and *Lactobacillus* were the predominant bacterial genera in the fecal microbiota of single-housed WT mice (Figure 5b), while *Prevotella* and *Lactobacillus* were the predominant genera in single-housed Ring1KO mice (Figure 5b). Co-housing with Ring1aKO mice for two weeks led to a considerable decrease in the proportion of *Akkermansia* and a dramatic increase in the proportion of *Prevotella* in WT mice (Figure 5b). Notably, significant differences were observed in the abundance of *Prevotella* and *Akkermansia* between single-housed WT and Ring1aKO mice, as well as between single-housed WT and WT-CH mice (Figure 5c). However, no significant difference in the abundance of *Lactobacillus* was observed between single-housed WT mice and Ring1aKO mice (Figure 5c). Considering our previous demonstration that Ring1a deficiency in immunocytes aggravates DSS-induced colitis, the altered intestinal bacterial composition in Ring1aKO mice is likely a consequence of intestinal immune dysfunction. IgA represents the most critical immunoglobulin that is transported and secreted into the gut lumen, thereby maintaining the regional intestinal ecosystems.^32,33^ To confirm whether the altered composition of intestinal bacteria in Ring1aKO mice is linked to changes in IgA production, we conducted mixed bone chimeric experiments to assess the impact of Ring1a deficiency on IgA production. The results revealed that Ring1a deficiency significantly reduced IgA^+^CD19^+^ B cells in Peyer’s patches and IgA^+^CD19 plasma cells in LP (Figures 5d,e). Moreover, the levels of IgA were significantly decreased in fecal samples and colon tissues of Ring1aKO mice compared to WT mice (Figures F and G). Hence, the decreased IgA production in Ring1aKO mice is likely responsible for the altered composition of intestinal bacteria.

(a) PCoA analysis of gut bacteria of single-housed and co-housed WT and Ring1aKO mice (*n* = 4-5). (b) Top 20 genus of gut bacteria of single-housed and co-housed WT and Ring1aKO mice. (c) Difference significance analysis of *Akkermansia* genus, *Prevotella* genus, and *Lactobacillus* genus in single-housed and co-housed WT and Ring1aKO mice (*n* = 4-5). In mixed bone marrow chimera mice, percentages of CD19^+^IgA^+^ cells in PPs (d) and percentages of CD19^−^IgA^+^ cells in LP (e) of CD45.1^+^ WT cells and CD45.2^+^ Ring1aKO cells (*n* = 5). IgA in feces (f) and colon tissues (g) of 8-week-old WT and Ring1aKO mice (*n* = 8). Data are shown as mean ± SD, representing one of at least three independent experiments. *P < .05, **P < .01.

### An exacerbation of colitis caused by Ring1a deficiency can be alleviated by metronidazole

**Figure 6.**
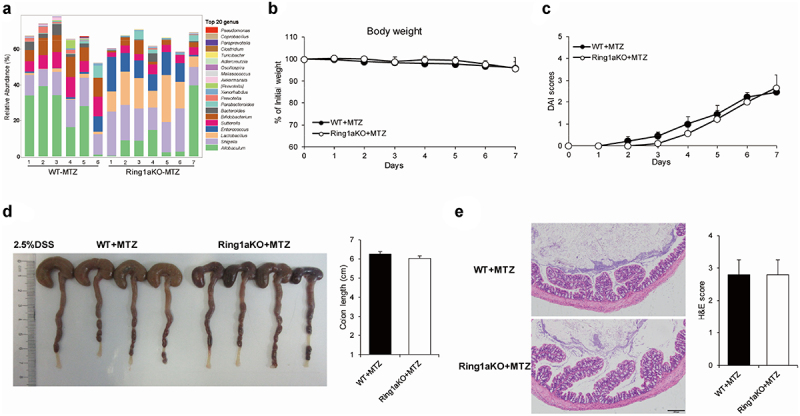
Metronidazole alleviated Ring1a deficiency caused exacerbation of colitis.

The *Prevotella* genus is a kind of gram-negative anaerobic bacteria that belongs to the *Bacteroidetes* phylum.^34^ To investigate whether depletion of the *Prevotella* genus can alleviate DSS-induced colitis in Ring1aKO mice, we treated WT and Ring1aKO mice with metronidazole, a compound known to deplete most gram-negative anaerobic bacteria. Following metronidazole treatment, the *Prevotella* genus was dramatically reduced in Ring1aKO mice, while a high proportion of the *Lactobacillus* genus was still observed in the feces of Ring1aKO mice (Figure 6a). Importantly, there was no difference in the severity of colitis between WT and Ring1aKO mice after metronidazole treatment. Ring1aKO mice exhibited similar weight loss, DAI scores, colon lengths, and histological inflammation compared to WT mice after metronidazole treatment (Figures 6b–e). These results suggest that the exacerbation of colitis in Ring1aKO mice is driven by colitogenic microbes and can be ameliorated using metronidazole.

(a) Top 20 genus of gut bacteria of metronidazole-treated WT and Ring1aKO mice (*n* = 6-7). The body weight (b) and DAI scores (c) of WT and Ring1aKO mice treatment with metronidazole in DSS-induced colitis (*n* = 5). (d) Colon lengths of WT and Ring1aKO mice treatment with metronidazole in DSS-induced colitis (*n* = 5). (e) H&E staining and H&E scores WT and Ring1aKO mice treatment with metronidazole in DSS-induced colitis (*n* = 5). Data are shown as mean ± SD, representing one of at least three independent experiments. *P < .05, **P < .01.

### Ring1a deficiency alters the immune homeostasis of intestinal LP

scRNA-seq has been widely used to reveal the heterogeneity and cellular composition of tissues in many human diseases and animal models. However, scRNA-seq has not yet been applied to investigate the cellular composition of the colon tissue in the DSS colitis model. In this study, we utilized scRNA-seq to gain insights into the detailed immunocyte composition of the colon tissue in DSS colitis and evaluate the effects of Ring1a deficiency on immunocyte populations. The analysis of CD45^+^ cells in the LP of WT and Ring1aKO mice was sorted and further analyzed by scRNA-seq.

**Figure 7.**
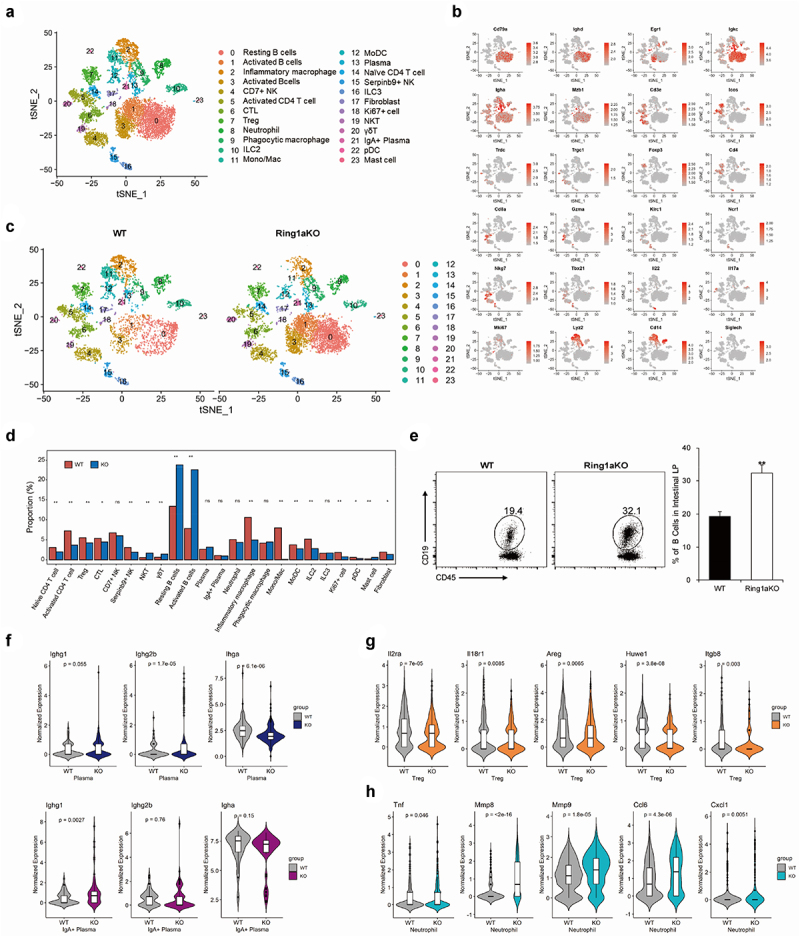
Ring1a deficiency altered immune landscape in intestinal LP.

CD45^+^ immunocytes in the intestinal LP exhibited high heterogeneity, as evidenced by the gene expression profiles and available cell markers from the CellMarker website and published literature. We identified 23 distinct cell types and states in the intestinal LP (Figure 7a and Supplementary Figure 2B). The putative 23 cell types contained nearly all kinds of immunocytes, including B cells, T cells, monocytes/macrophages, neutrophils, innate lymphoid cells (ILCs), NK cells, NKT cells, and γδT cells (Figure 7a,b), which indicates the involvement of nearly all immune cell populations in the pathogenesis of DSS colitis. Furthermore, Ring1a deficiency resulted in remarkable alterations in the proportions of several immune cell groups, including B cells, monocytes/macrophages, ILCs, NKT cells, and γδT cells (Figure 7c,d). Notably, Ring1a deficiency led to a notable increase in the percentage of B cells (groups 0, 1, and 3), a finding that was further validated using fluorescence activated cell sorting (FACS) (Figure 7e). Subsequent *t-SNE* clustering analysis of B cells revealed five distinct subclusters. Notably, compared to WT mice, the percentages of IgA^+^ plasma cells in Ring1aKO mice decreased (Supplementary Figure S3), consistent with the aforementioned results obtained from the mixed bone marrow chimera experiment. In group 13 (plasma cells), we also found that *Igha* expression was significantly decreased and *Ighg2b* expression was significantly increased in Ring1KO mice (Figure 7f). Additionally, in group 21 (IgA^+^ plasma cells), the expression of *Ighg1* was boosted considerably in Ring1aKO mice (Figure 7f), indicating a tendency for increased production of IgG1 by plasma cells in Ring1aKO mice. Moreover, Ring1a deficiency altered the proportions of monocytes/macrophages (groups 2, 9, and 11), especially group 11, which exhibited high expression of CXCL9 and CXCL10 (Supplementary Figure S2). Group 11 almost disappeared in Ring1aKO mice (Figures 7c,d). Additionally, pro-inflammatory IL-17^+^ γδT cells and cytotoxic γδ/CD8 Trm cells were increased, while ILC2s were decreased in Ring1aKO mice (Figures 7c,d).

\(a) *tSNE* plot of 23 identified cellular populations in intestinal LP. (b) FeaturePlots of classical cell markers of lymphocytes, γδ T cells, monocytes, NK cells, macrophages, and ILC. (c) *tSNE* plot of 23 identified cellular populations in the intestinal of WT and Ring1aKO mice. (d) Percentages of 23 identified cellular populations in the intestinal of WT and Ring1aKO mice. (e) Percentages of CD19^+^ B cells in intestinal LP of WT and Ring1aKO mice. (f) Expressions of *Ighg1*, *Ighg2b*, and *Igha* in plasma and IgA^+^ plasma of WT and Ring1aKO mice. (g) Expressions of *Il2ra*, *Areg*, *Huwel*, *Il18r1*, and *Itgb8* in Treg cells of WT and Ring1aKO mice. (h) Expressions of *Mmp8*, *Mmp9*, *Ccl6*, *Il18r1*, *Tnf*, and *Cxcl1* in Treg cells of WT and Ring1aKO mice.

Treg cells in the intestinal microenvironment are important to maintain intestinal homeostasis by controlling inflammation.^35^ Comparatively, the proportion of Treg cells slightly decreased in Ring1aKO mice. Notably, the expressions of suppression function-associated genes, such as *Areg*, *Il2ra*, *Il18r1*, *Huwe1*, and *Itgb8*, significantly reduced in Treg cells of Ring1aKO mice than those of WT mice (Figure 7g), indicating a diminished anti-inflammatory function of Treg cells in Ring1aKO mice. Additionally, although there was no difference in the percentage of neutrophils between WT and Ring1aKO mice, pro-inflammatory function-associated genes (*Mmp8*, *Mmp9*, *Cxcl1*, *Ccl6*, and *Tnf*) were significantly upregulated in neutrophils from Ring1aKO mice (Figure 7h), suggesting an enhanced pro-inflammatory function of neutrophils in Ring1aKO mice.

## Discussion

IBD, encompassing CD and UC, represents a diverse group of chronic inflammatory disorders. Despite extensive research employing advanced technologies and experimental models, the precise pathogenesis of IBD remains elusive. The current understanding of IBD etiology involves interweaving host genetics, host immunity, the gut microbiome, and environmental exposures.^36^ Although over 200 loci, including *NOD2* and *PTPN22*, have been identified as IBD risk variants, they account for only 8%–13% of disease susceptibility.^36,37^ Consequently, it is crucial to explore potential novel candidate risk genes for IBD and unravel the complex interactions driving the disease.

The gastrointestinal tract houses a complex network of interactions between the microbiome, epithelium, and immune cells.^38^ However, the mechanisms underlying the involvement and interconnections of these disease drivers remain poorly understood. As the catalytic core of PRC1, Ring1a has been implicated in gene expression regulation through H2AK119ub1 catalysis and chromatin remodeling.^12–14^ However, its functions in immune cells and immune-associated IBD have not been extensively studied. In this study, we identified the PcG protein Ring1a as a crucial player in IBD pathogenesis by regulating both host immunity and intestinal microbiota, thus potentially serving as a new risk gene for IBD. Notably, In DSS-induced colitis, Ring1a deficiency significantly exacerbated the symptoms in the DSS-induced colitis model of the disease. We employed Ring1aKO mice, which encompass a whole-body knockout of Ring1a, to investigate the role of Ring1a deficiency in immune cells in the exacerbation of DSS colitis. To further elucidate this relationship, we constructed bone marrow chimeras. The findings revealed that Ring1a deficiency in immune cells intrinsically aggravated DSS colitis.

Additionally, we also found that co-housing Ring1aKO mice with WT mice for two weeks led to aggravated DSS colitis in the WT mice, suggesting that Ring1a deficiency increased the pathogenicity and transmissibility of the intestinal microbiota. Importantly, the effect of the transferable pathogenic intestinal microbiota from Ring1aKO mice to co-housed WT mice persisted for at least one month. When Ring1aKO mice were co-housed with WT mice for more than two weeks, regardless of subsequent separation, the pathogenic intestinal microbiota of Ring1aKO mice dominated and induced severe colitis in the WT mice.

By employing antibiotics to selectively target bacteria or fungi, we found that it is gut bacteria, rather than fungi, contribute to the development of severe colitis in Ring1aKO mice. The *Prevotella* genus has been reported to be associated with several autoimmune diseases, including insulin resistance and diabetes, arthritis and intestinal inflammation.^39^ Several studies have indicated that a *Prevotella*-dominated microbiome may promote inflammation, with the *Prevotella* genus implicated as a potential pathogenic bacterium in IBD. Genetically altered mice with *Prevotella*-dominated gut microbiota or oral gavage of *Prevotella* intestinalis have shown increased susceptibility to colitis. NLRP6 deficiency in mouse colonic epithelial cells exacerbates DSS-induced colitis and promotes the expansion of *Prevotellaceae* and TM7.^27^ Furthermore, colonization of mice with a novel *Prevotella* genus species from NLRP6-deficient mice exacerbates DSS-induced colitis via reducing IL-18 production.^39^ However, another study showed that caspase-11 deficiency exacerbates DSS-induced colitis independently of gut microbiota alterations, with a considerable reduction in *Prevotella* species.^40^ Our 16s rRNA sequencing results revealed that Ring1a deficiency dramatically altered the composition of intestinal bacteria, leading to a remarkable expansion of the *Prevotella* genus and a decrease in the *Akkermansia* genus. Importantly, the *Prevotella* and *Akkermansia* genera differed considerably between single-housed WT and Ring1aKO groups, as well as between WT and WT-CH groups. Moreover, the *Prevotella* genus was significantly reduced in metronidazole-treated Ring1aKO mice. These findings suggest that the *Prevotella* genus may be the main colitogenic bacteria contributing to the exacerbated colitis observed in Ring1aKO mice and that metronidazole could alleviate colitis exacerbation in Ring1aKO mice by reducing the *Prevotella* genus. However, further investigation is warranted to establish direct evidence regarding the role of the *Prevotella* genus in colitis.

The *Akkermansia* genus has garnered attention as a promising next-generation probiotic due to its therapeutic potential in metabolic diseases.^41^ Consistent with our results, numerous studies have reported a negative correlation between *Akkermansia muciniphila* and IBD, which exhibit a marked decrease in the abundance of *Akkermansia muciniphila*. However, several studies also showed the controversial role of *Akkermansia muciniphila* in mouse models of IBD. *Akkermansia muciniphila* enrichment was observed in *Nlrp6* and *Il10* double-deficient mice and can induce intestinal inflammation in germ-free and specific-pathogen-free *Il10*-deficient mice.^42^ Additionally, 1,25(OH)2D3 deficiency has been linked to increased *Akkermansia muciniphila* and colon inflammation phenotypes in Cyp27b1 knockout mice.^43^ These seemingly contradictory effects may be attributed to specific strain differences, necessitating further studies to elucidate their roles in IBD.

With the emergence and widespread adoption of scRNA-seq technology, several studies have utilized this advanced approach to unravel the complex pathogenesis of human IBD.^44–46^ However, to date, no study has applied single-cell transcriptome technology to investigate the immune landscape in the DSS-induced colitis model, which closely resembles the pathological features of human IBD. Although FACS has demonstrated immune cell infiltration and activation in DSS colitis,^47^ technical limitations have hindered a comprehensive understanding of the immune landscape in this model. In this study, we first investigated the immune microenvironment of intestinal LP in DSS colitis using single-cell transcriptome sequencing technology. In the sorted CD45^+^ immunocytes of intestinal LP, we identified 23 distinct clusters. The immunocytes in LP exhibited remarkable heterogeneity, representing various immune cell populations, including T cells, B cells, monocytes/macrophages, DC, NK cells, NKT cells, and ILCs, all of which participated in the intestinal immune microenvironment of DSS colitis. ILC3s are conventionally believed to play important roles in maintaining intestinal homeostasis.^38,48^ Unexpectedly, we observed a higher abundance of ILC2s compared to ILC3s in the intestinal LP, suggesting a potential involvement of ILC2s in the pathogenesis of IBD.

To further explore the immune mechanisms underlying the exacerbation of DSS colitis caused by Ring1a deficiency, we also sorted and analyzed CD45^+^ immunocytes in the intestinal LP of Ring1aKO mice using scRNA-seq. Remarkably, Ring1a deficiency significantly altered the intestinal bacteria even before the implementation of the DSS-induced colitis model. The observed intestinal immune landscape in Ring1aKO mice reflects the integrated effects of Ring1a deficiency in immunocytes and the altered gut bacteria induced by the deficiency.

Compared to WT mice, Ring1a deficiency significantly increased the proportion of B cells, including resting and activated B cells. The functional roles of B cells in IBD remain unclear, with conflicting findings reported in the literature. For instance, Do et al. demonstrated the increased infiltration of B lymphocytes within the inflamed LP in DSS colitis^47^ and posited that the HDAC6 inhibitor BML-281 selectively attenuated B cell infiltration into the LP, potentially contributing to the amelioration of colonic inflammation.^47^ Conversely, Wang et al. found that DSS-treated mice exhibited more severe colitis in the absence of B cells, while the adoptive transfer of B cells attenuated the disease.^49^ IgA-producing plasma cells are essential to maintaining intestinal homeostasis in the normal gut. However, there is a shift in the mucosal immune system from a predominance of IgA-producing plasma cells to an abundance of plasma cells dedicated to IgG production in IBD,^50^ a characteristic that we also observed in Ring1KO mice.

Our analysis revealed a reduction in ILC2s in Ring1aKO mice, suggesting a protective role for ILC2s in DSS colitis. Several studies have detected ILC2s in the intestines of IBD patients and demonstrated their protective roles in colitis models.^51,52^ For instance, You et al. transferred purified ILC2s to Rag1^−/−^ mice and found that they could alleviate DSS-induced acute innate colitis, potentially by promoting M2 macrophage polarization.^53^ Similarly, the adoptive transfer of IL-33-expanded ILC2s to DSS-treated mice significantly reduced colonic inflammation compared to DSS control mice.^54^ Moreover, Uddin et al. identified the IL-33-ILC2s pathway as an important host defense mechanism against amebic colitis, as treatment with IL-33 protected Rag2^−/−^ mice from amebic colitis by increasing recruitment of ILC2s.^55^ However, Qiu et al. reported that the IL-33-ILC2s axis may have pathogenic effects in IBD, as they found that IL-33 deficiency protected mice from DSS-induced experimental colitis by suppressing ILC2s and Th17 cell responses.^56^ Further studies are warranted to fully elucidate and confirm the functions of ILC2s in IBD, and targeting ILC2s could hold promise as a novel therapeutic target for IBD treatment.

Furthermore, the proportions of IL-17A^+^ γδ T cells and a cluster characterized as cytotoxic γδ/CD8 Trm cells were significantly increased in the intestinal LP of Ring1aKO mice. IL-17^+^ γδ T cells are known to contribute to protective anti-microbial responses and are implicated in pathogenic inflammation at barrier sites. For instance, Do et al. revealed that IL-17^+^ γδ T cells play a crucial role in enhancing *in vivo* Th17 differentiation and T cell-mediated colitis. They found that TCRβδ^−/−^ mice were resistant to colitis, and cotransfer of IL-17^+^ γδ T cells with CD4 T cells was sufficient to enhance Th17 differentiation and induce colitis in TCRβδ^−/−^ recipients.^57^ In EAE, IL-17-producing IL23R^+^ γδ T cells were found to accumulate in the CNS and inhibit the suppressor activity of Treg cells.^58^ Consistently, in our study, we observed a remarkable decrease in the suppressive function of Treg cells in Ring1aKO mice, as evidenced by marked decreased expressions of genes including *Areg*, *IL2RA*, *Il18r1*, *Huwe1*, and *Itgb8*, all of which have been associated with the suppressive function of Treg cells.^59–63^ The cluster of IL-17^+^ γδ T cells also exhibited a high level of Cxcr6 expression, which may be associated with their migration to a specific location.

In the present study, we identified a cluster of cytotoxic γδ/CD8 Trm cells that showed a significant increase in Ring1aKO mice. The cluster of cytotoxic γδ/CD8 Trm cells highly expressed cytotoxic markers such as Gzma, Gzmb, Ccl5, and Klrd1, Itgae/CD103, indicating their potential cytotoxicity against epithelial cells and their ability to recruit inflammatory cell types that promote tissue destruction. Notably, Hu et al. discovered a cluster of intraepithelial γδ T cells with a similar gene expression profiles to the cytotoxic γδ/CD8 Trm cells identified in the intestinal LP. They found that these intraepithelial γδ T cells facilitated pathological epithelial cell shedding via CD103-mediated granzyme release,^64^ thereby promoting transient gaps in the epithelial barrier and increased intestinal permeability.^65^ Similarly, Do et al. also identified a CD103^+^α4β7^high^ γδ T subset in mesenteric lymph nodes that highly expressed *Gzma*, *Gzmb*, *Klrd1*, and *Ccl5*, which promoted intestinal inflammation and enhanced disease severity.^66^ Gzma, a pro-inflammatory protease involved in the regulation of the inflammatory response in intestinal hemostasis, exerts a pivotal role. Santiago et al. found that Gzma deficiency reduced gut inflammation and colorectal cancer development.^67^

In summary, our data provide evidence for the role of the PcG protein E3 ubiquitin ligase Ring1a in colitis. Ring1a plays a protective role in colitis by synthetically regulating immune cells and the gut microbiota. Ring1a deficiency exacerbates DSS colitis due to its deficiency in the immune system. Meanwhile, Ring1a deficiency dramatically alters the community of intestinal bacteria, leading to a *Prevotella* genus-dominated pathogenesis microenvironment that can be horizontally transmissible to co-housed WT mice and aggravates DSS colitis. We also find that Ring1a deficiency causes microbiota disorder potentially due to decreased IgA and prove that Ring1a deficiency leads to exacerbation of colitis that can be alleviated by metronidazole treatment. Furthermore, we describe the immune landscapes of mouse DSS colitis for the first time and reveal that Ring1a deficiency alters intestinal immune homeostasis. Our results suggest that Ring1a may be a new potential candidate risk gene for colitis, and metronidazole may treat *Prevotella* genus-dominated colitis clinically.

## Supplementary Material

Supplemental Material

## Data Availability

The scRNA-seq data has been submitted to the GEO database (GSE210866, https://www.ncbi.nlm.nih.gov/geo/query/acc.cgi?acc=GSE210866). Other data underlying this article are available in the article and supplementary materials.

## Abbreviations

CD: Crohn’s disease
DAI: Disease activity index
DSS: Dextran sodium sulfate
FACS: Fluorescence activated cell sorting
H&E: Hematoxylin and eosin
H2AK119ub1: Monoubiquitylation of histone H2A at lysine 119
IBD: Inflammatory bowel disease
ILCs: Innate lymphoid cells
LP: Lamina propria
PcG: Polycomb group
PRC: Polycomb repressive complexes
scRNA-seq: Single-cell RNA sequencing
Ring1aKO: Ring1a knockout
UC: Ulcerative colitis
WT: Wild type
7AAD: 7-aminoactinomycin
