# Supplementary material for: Ring1a protects against colitis through regulating mucosal immune system and colonic microbial ecology
Source: Gut Microbes. 2023 Sep 1;15(2):2251646. doi: 10.1080/19490976.2023.2251646 (PMC10478745; doi:10.1080/19490976.2023.2251646)
Supplement: Supplemental Material [file KGMI_A_2251646_SM2169.docx]

**Supplementary figures**

**Supplementary Fig 1**

**Supplementary Fig 1. Initial weights of WT and Ring1aKO mice before conducted DSS colitis.**

**Supplementary Fig 2**


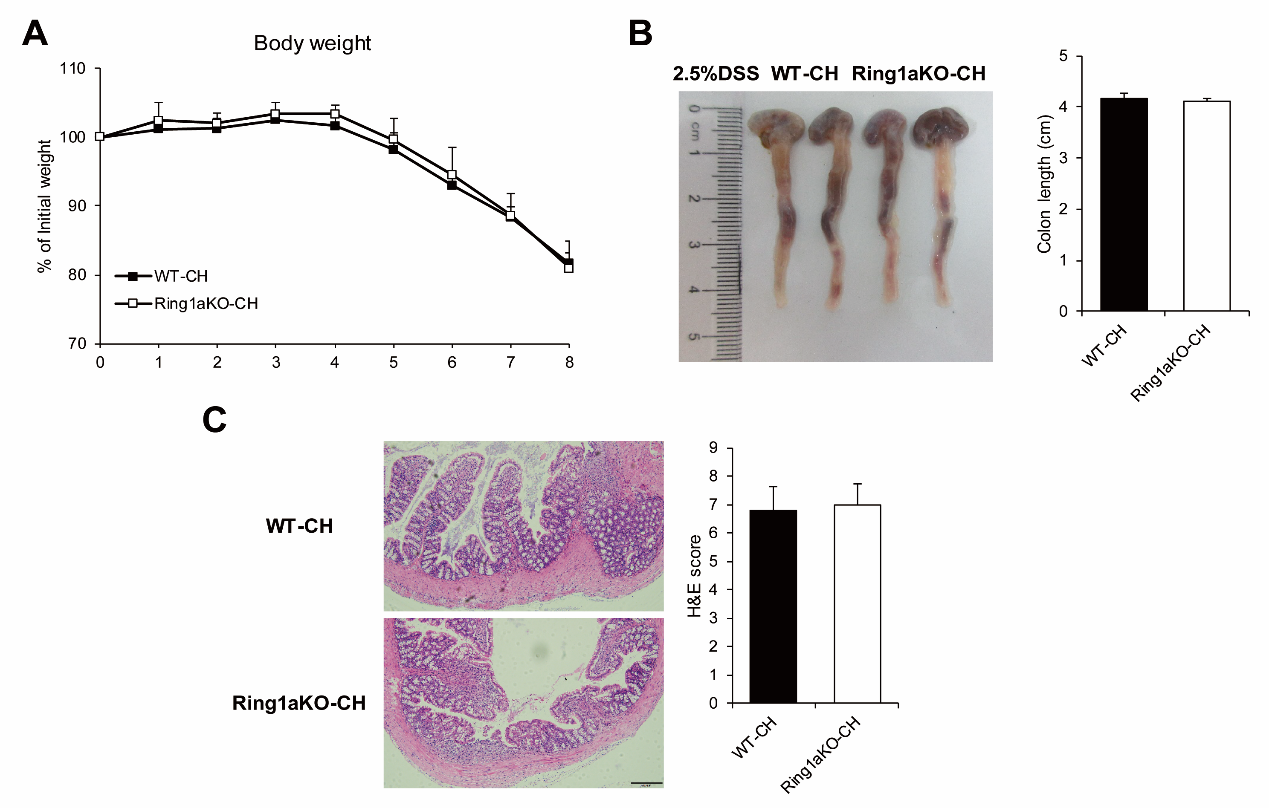


**Supplementary Fig 2. Ring1a deficiency caused exacerbation of colitis and was transferable to co-housed WT mice.**

(**A**) The body weight of co-housed WT and Ring1aKO mice in DSS-induced colitis. (**B**) Colon lengths of co-housed WT and Ring1aKO mice in DSS-induced colitis. (**C**) H&E staining and H&E scores of co-housed WT and Ring1aKO mice in DSS-induced colitis. Data are shown as mean ± SD (n=3), representing one of at least two independent experiments. **P* < 0.05, ***P* < 0.01.

**Supplementary Fig 3**


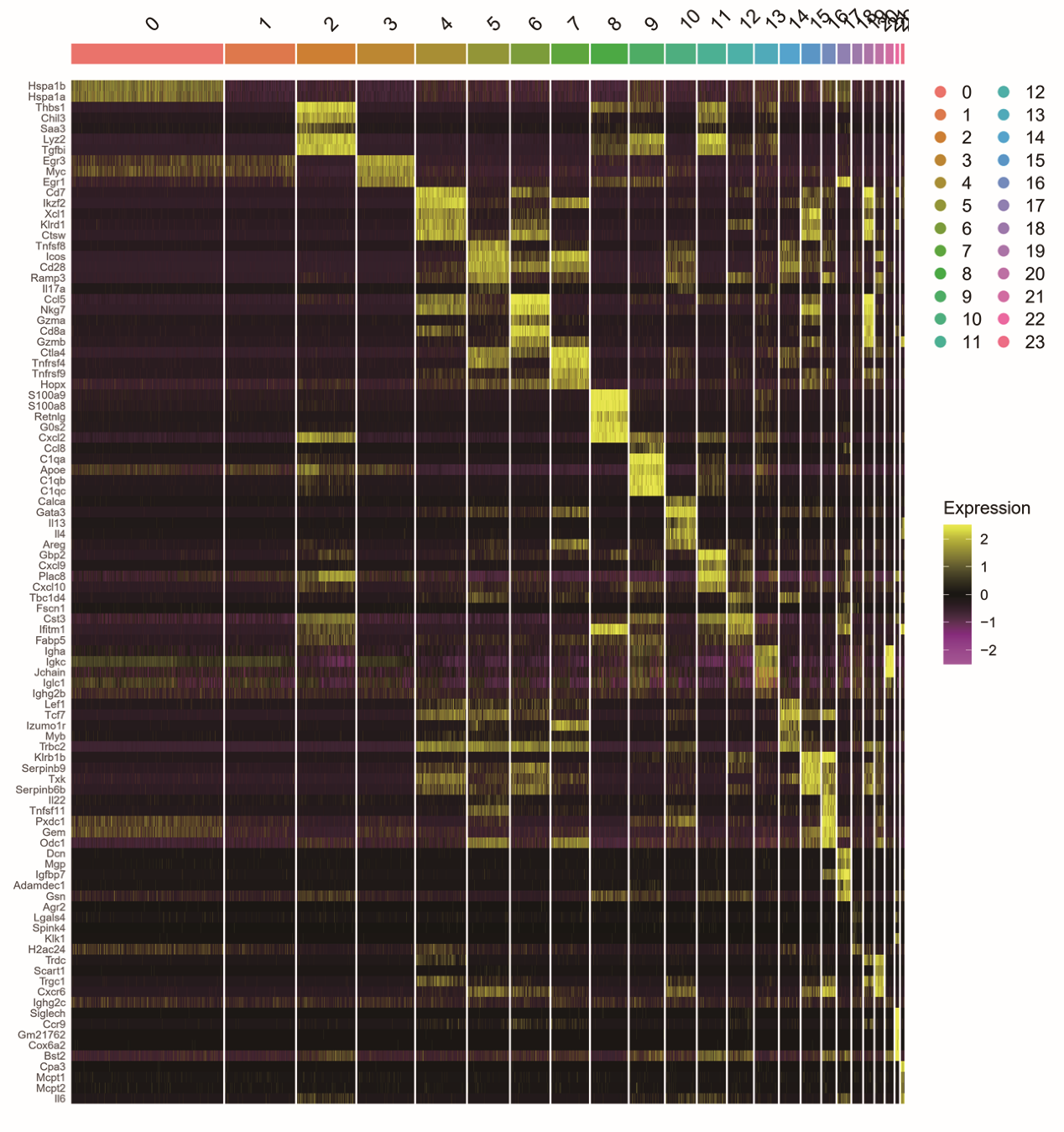


**Supplementary Fig 3. Gene expression profile of immune cell populations in scRNA-seq.**

*tSNE* visualization of WT-Ring1A integrated scRNA-seq was divided into 23 color-labeled groups at 0.7 resolution. The color scale indicates the level of gene expression. Yellow represents relatively high expression and purple represents relatively low expression.

**Supplementary Fig 4**

**
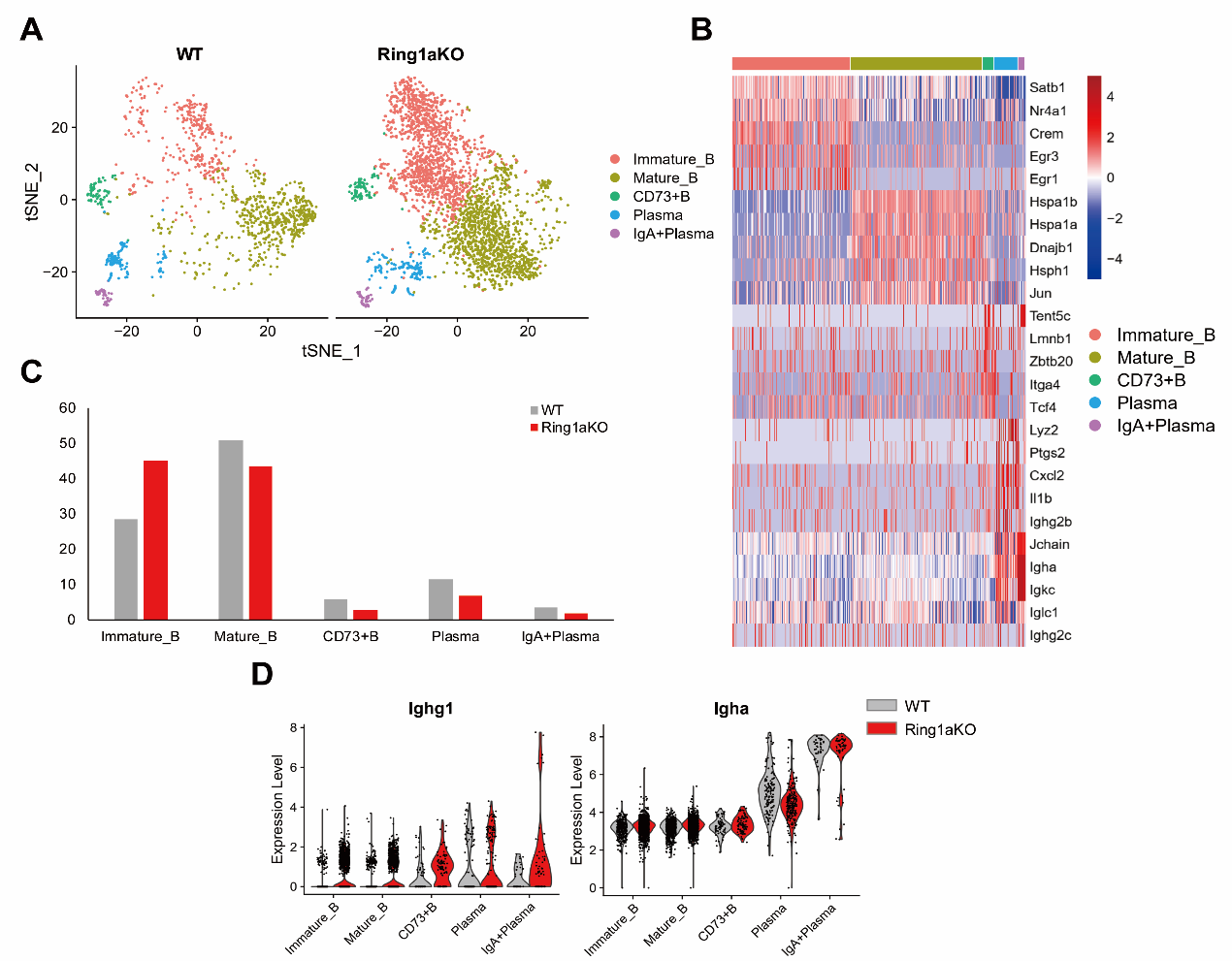
**

**Supplementary Fig 4. Ring1a deficiency altered B cell populations in intestinal LP.**

(**A**) *tSNE* plot of five identified B cell populations in intestinal LP of WT and Ring1aKO mice. Red represents relatively high expression and blue represents relatively low expression. (**B**) Gene expression profile of five identified B cell populations. (**C**) Percentages of five identified B cell populations in the intestinal of WT and Ring1aKO mice. (**D**) Expressions of *Ighg1* and *Igha* in five identified B cell populations of WT and Ring1aKO mice.
